# Transcriptomic analysis reveals specific osmoregulatory adaptive responses in gill mitochondria-rich cells and pavement cells of the Japanese eel

**DOI:** 10.1186/s12864-015-2271-0

**Published:** 2015-12-18

**Authors:** Keng Po Lai, Jing-Woei Li, Je Gu, Ting-Fung Chan, William Ka Fai Tse, Chris Kong Chu Wong

**Affiliations:** School of Biological Sciences, Kadoorie Biological Sciences Building, The University of Hong Kong, Pokfulam Road, Pok Fu Lam, Hong Kong; State Key Laboratory of Agrobiotechnology, The Chinese University of Hong Kong, Shatin, Hong Kong; Faculty of Medicine, The Chinese University of Hong Kong, Shatin, Hong Kong; Department of Biology, Hong Kong Baptist University, Kowloon Tong, Hong Kong; Croucher Institute for Environmental Sciences, Hong Kong Baptist University, Pok Fu Lam, Hong Kong

**Keywords:** Next generation sequencing, Osmotic stress, Fish

## Abstract

**Background:**

Homeostasis of ions and water is important for the maintenance of cellular functions. The regulation of the homeostasis is particularly important in euryhaline fish that migrate between freshwater (FW) and seawater (SW) environments. The fish gill, the major tissue that forms an interface separating the extracellular fluids and external water environment, has an effective transport system to maintain and regulate a constant body osmolality. In fish gills, the two major epithelial cells, pavement cells (PVCs) and mitochondria-rich cells (MRCs), are known to play key and complementary roles in ion transport at the interface. Discovering the robust mechanisms underlying the two cell types’ response to osmotic stress would benefit our understanding of the fundamental mechanism allowing PVCs and MRCs to handle osmotic stress. Owing to the limited genomic data available on estuarine species, existing knowledge in this area is slim. In this study, transcriptome analyses were conducted using PVCs and MRCs isolated from Japanese eels adapted to FW or SW environments to provide a genome-wide molecular study to unravel the fundamental processes at work.

**Results:**

The study identified more than 12,000 transcripts in the gill cells. Interestingly, remarkable differential expressed genes (DEGs) were identified in PVCs (970 transcripts) instead of MRCs (400 transcripts) in gills of fish adapted to FW or SW. Since PVCs cover more than 90 % of the gill epithelial surface, the greater change in gene expression patterns in PVCs in response to external osmolality is anticipated. In the integrity pathway analysis, 19 common biological functions were identified in PVCs and MRCs. In the enriched signaling pathways analysis, most pathways differed between PVCs and MRCs; 14 enriched pathways were identified in PVCs and 12 in MRCs. The results suggest that the osmoregulatory responses in PVCs and MRCs are cell-type specific, which supports the complementary functions of the cells in osmoregulation.

**Conclusions:**

This is the first study to provide transcriptomic analysis of PVCs and MRCs in gills of eels adapted to FW or SW environments. It describes the cell-type specific transcriptomic network in different tonicity. The findings consolidate the known osmoregulatory pathways and provide molecular insight in osmoregulation. The presented data will be useful for researchers to select their targets for further studies.

**Electronic supplementary material:**

The online version of this article (doi:10.1186/s12864-015-2271-0) contains supplementary material, which is available to authorized users.

## Background

Ion-osmoregulation is vital for the maintenance of tissue and cellular functions. It is important not only to define intracellular osmolality and cell shape, but it is also crucial for guiding many cellular functions, including transepithelial transport, cellular metabolism, and cell migration [1]. The fundamental mechanism of the regulation is highly conserved in metazoan; however, plasticity and efficiency of the regulation may vary with respect to the evolutionary development of animals during adaptation. Aquatic organisms are known to have well-developed osmoregulatory apparatus to regulate fluid and ion transport when faced with changes in water osmolality and to maintain a constant body osmolality. In euryhaline fish (i.e., salmon and eels), those that are naturally exposed to waters of varying salinity during their life cycles, maintaining a constant plasma osmolality when faced with an osmotic challenge has become a habitual and normal process. The gills of euryhaline fish are recognized as highly efficient ion-osmoregulatory apparatus evolved to adapt to large changes in external osmolality. Therefore, it is an excellent tissue model to reveal the fundamental mechanism associated with plasticity in ion-osmoregulatory processes.

There are two major types of gill cells, the pavement cells (PVCs) and the mitochondria-rich cells (MRCs), which play key roles in ion-osmoregulation, namely sodium (Na^+^), chloride (Cl^−^), and calcium (Ca^2+^) transport, and acid–base balance. Early studies using electron microscopic analysis reported remodeling of gill tissues and identified changes in the relative abundance of PVCs and MRCs in fish acclimated to waters of different salinities [2, 3]. Electrophysiological studies using an alternate inner opercular membrane tissue model have indirectly demonstrated the ion transport functions of gill cells [4, 5]. Later studies, using both histochemical and nucleic acid analyses, showed the spatiotemporal expression of different ion transporters in PVCs and MRCs of fish acclimated to freshwater (FW) or seawater (SW) environments, with the aim of delineating possible functions [6, 7]. The techniques of subtractive hybridization, microarray, and rapid amplification of cDNA ends (RACEs) have been applied to reveal differential gene expressions in gill tissues collected from FW- or SW-adapted fish [8–10]. However, the significance of the studies was hindered by the limitation in identifying new transcripts with low sequence similarities to the existing sequence databases. Nonetheless, different working models to describe the ion transport activities of the two cell types have been proposed and described in recent review papers [1, 11]. Basically, the growth and differentiation of gill PVCs and MRCs were found to respond differently in FW and SW environments and the cells played different but complementary roles in ion transport [12–14]. Findings during the past decades have provided unequivocal evidence strengthening our understanding of the fundamental mechanism of the cells in ion-osmoregulation; however, the scope of the studies has largely been limited by the availability of molecular tools (i.e., genomic database, homogeneous antibody) in the fish species. Therefore, advancement of the understanding of the fundamental molecular mechanisms undertaken in the two cell types has been challenging. Knowledge gaps in differential responses of PVCs and MRCs to osmotic stress have been identified. In this study, we hypothesized that PVCs and MRCs adopt different molecular mechanisms against an osmotic challenge to gain functional plasticity with respect to their roles and anatomical localizations. We aimed to conduct transcriptomic analysis of isolated PVCs and MRCs from both FW- and SW-adapted eels, followed by bioinformatics analysis and data verification, to decipher the molecular processes undertaken in these two type of cells.

## Results and Discussion

Ion-osmoregulation is one of the key physiological processes essential for the maintenance of a consistent internal environment. Euryhaline fish are known to be an important model in the investigation of this fundamental and evolutionarily conserved process. In 2012, the first draft genome sequence of the Japanese eel was reported, in which a total assembled genome size was 1.15 Gbp and an N50 of 52,849 bp was produced using the Illumina platform [15]. Moreover, transcriptomic analysis of juvenile European eels or specific body tissues (i.e., gills and corpuscles of the Stannius gland) of Japanese eels were published using the Roche 454 or Illumina MiSeq platform [16–18]. In the gill transcriptome of the Japanese eel, 11,033 transcripts were identified in fish adapted to FW or SW conditions [17]. In a recent study using tilapia (*Oreochromis mossambicus*) as the model fish with the 454 platform, more than 5,000 annotated gill transcripts were reported [19]. The transcriptomic analysis of fish gills summarized differential expressed genes in both hypo- and hyper-osmoregulation. However, the molecular responses in the gill PVCs and MRCs in relation to their functional phenotypes are still unknown.

**Fig. 1:**
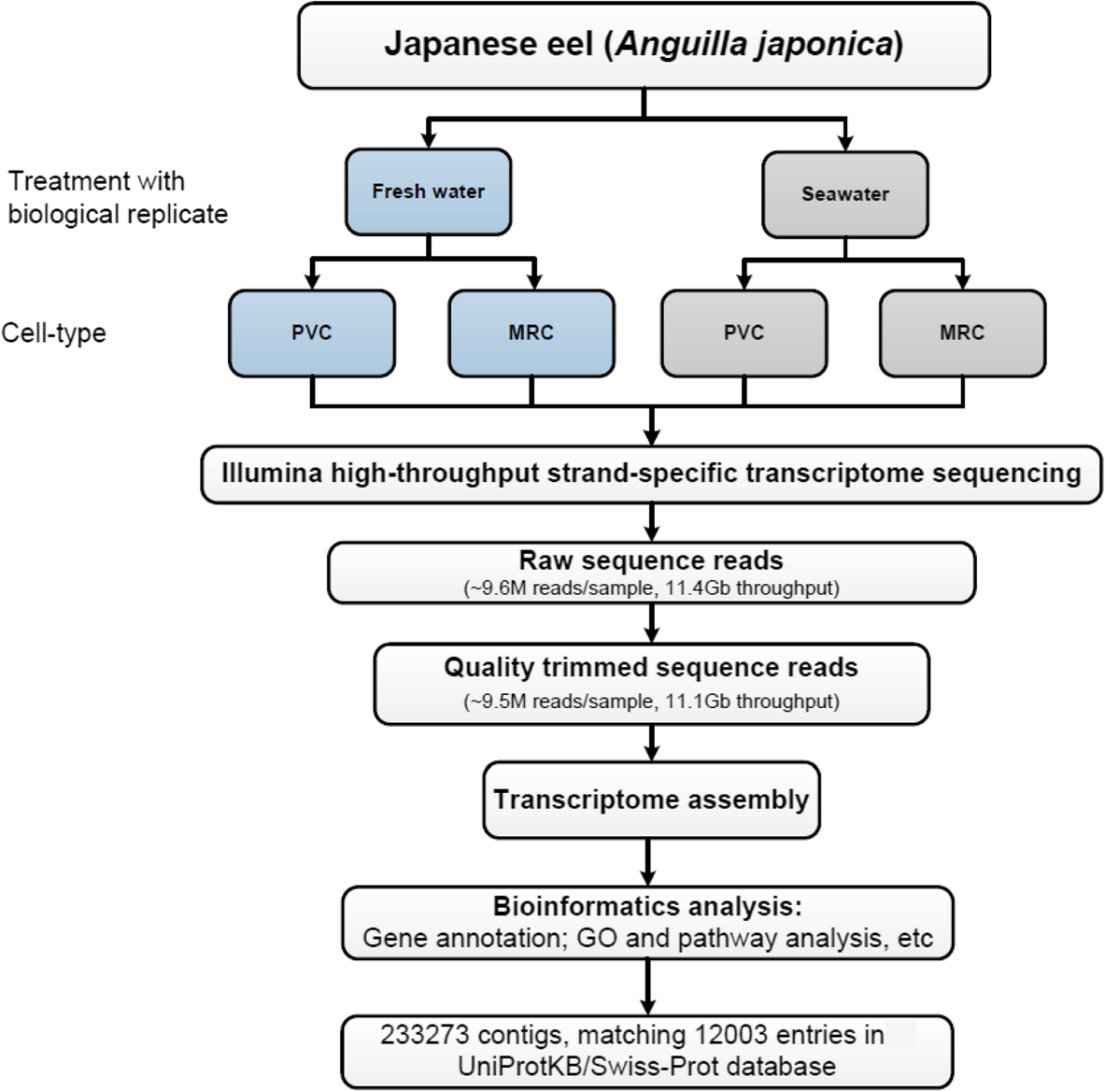
Workflow of Illumina deep sequencing and bioinformatics analyses. It includes sample preparation, cDNA library construction, Illumina sequencing, and data analyses including transcriptome assembly, BLAST search, GO annotation, IPA analysis, and gene expression analysis

In this study, we conducted transcriptomic analysis to identify differential gene expression profiles in gill PVCs and MRCs from Japanese eels adapted to FW or SW conditions. To move beyond previous transcriptomic analyses of fish gills, we used our established gill cell isolation protocol, a discontinuous Percoll gradient centrifugation, to enrich the two cell types, with cell purity greater than 85 % [20, 21]. The method was validated by other research groups and applied for functional characterization of the individual cell types in other fish models [22, 23]. Furthermore, flow cytometry and electronic microscopy characterizing the two types of cells had been performed in our group to confirm the fraction specificity [24, 25]. After RNA sequencing of the isolated cell types, we profiled and compared the expression levels of transcripts derived from the four groups of samples, (i) FW PVCs, (ii) SW PVCs, (iii) FW MRCs, and (iv) SW MRCs, using the Database for Annotation, Visualization, and Integrated Discovery (DAVID) and Ingenuity Pathway Analysis (IPA) bioinformatics analysis. The general workflow is shown in Fig. 1. The results, presented here for the first time, demonstrated the genome-wide molecular regulatory networks undertaken in gill PVCs and MRCs in response to osmotic challenges.

### The transcriptome of gill PVCs and MRCs generated by the Mi-Seq platform

**Fig. 2:**
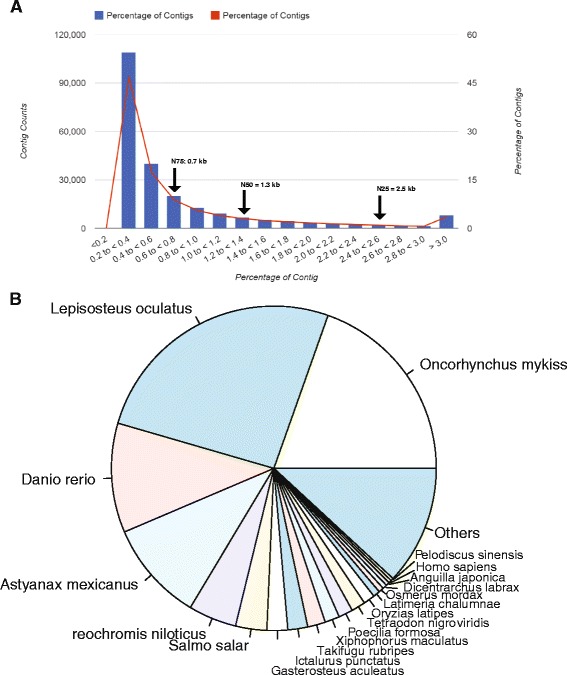
Summary of the *de novo* assembly of the eel gill PVCs and MRCs transcriptome. **a** Distribution of the assembled contigs with different contig length (kb). Included are contig lengths for N-25, N-50, and N-75. **b** Species distribution of matched genes, with the highest similarity to *Lepisosteus oculatus*

Sequencing libraries were prepared by enriched populations of PVCs and MRCs in both FW and SW conditions. Using the Illumina MiSeq platform, 9.5 M reads per sample, with a total of 11.1 Gb throughput, were obtained. Among 233,273 assembled contigs, a total of 12,003 transcripts were identified in reference to the UniProtKB/Swiss-Prot database (Fig. 1; Additional file 1). The distribution of contig-length ranged from 200 to 16,333 bp and the average contig-length was 783 bp. The N75, N50, and N25 were 0.7 kb, 1.3 kb, and 2.5 kb, respectively (Fig. 2a). Regarding the taxonomic distribution of the identified 12,003 genes, 25.9 % of the genes showed the highest similarities to *Lepisosteus oculatus*, followed by *Oncorhynchus mykiss* (19.6 %) and *Danio rerio* (10.9 %) (Fig. 2b; Additional file 2). The low percentage of the matched genes from *Anguilla* species (0.3 % in this study) is due to the lack of qualified genome databases and limited protein sequences of the fish species deposited in the UniProt database [18].

**Fig. 3:**
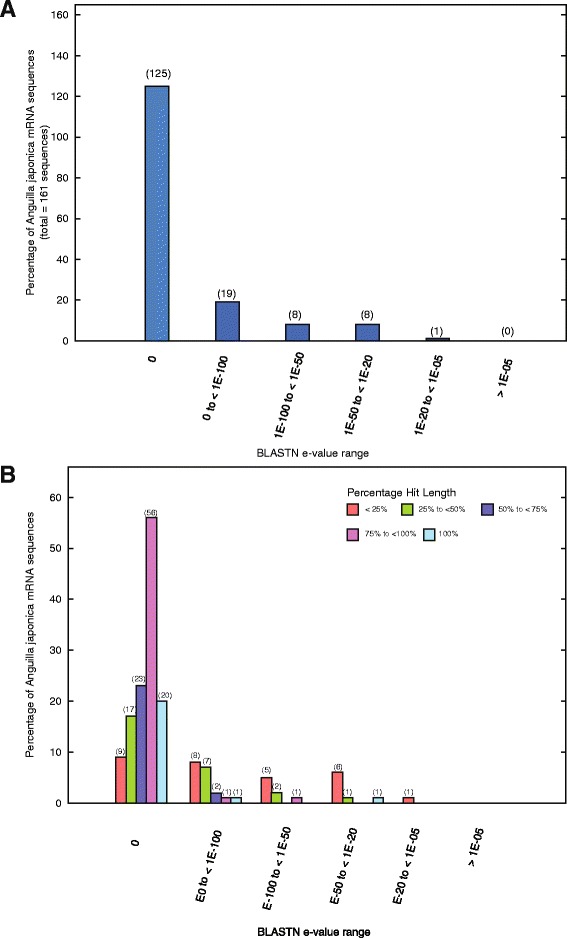
Evaluation of eel gill cell transcriptome. **a** Percentage distribution (number) of the 161 Sanger sequences with respective best hit grouped within different BLASTN E-value range. **b** Percentage distribution (number) of the 161 Sanger sequence with different full-length sizes (bp) grouped within different BLASTN E-value range

In comparing the cell-type specific (PVCs v. MRCs) transcriptome data with our previous published whole-gill transcriptome [17], 7,034 transcripts were found to be commonly annotated (Additional file 3). Nine hundred eighty-three newly annotated transcripts were first identified in this study. We matched our data to the National Center for Biotechnology Information (NCBI) database, in which 259 *Anguilla japonica* transcripts with complete coding sequences were available. In BLASTN, we found that 161 (62 %) deposited sequences matched our unified PVC/MRC transcript data. Among those, 125 of 161 sequences showed excellent hits with E-values equal to zero, while another 27 sequences had E-values less than 1E-50 (Fig. 3a). The coverage of the sequence lengths is shown in Fig. 3b. The data showed that the majority of the assembled sequences exhibited excellent hits (E-value = 0), and most of them were covered by at least 75 % mRNA sequence with respect to the full-length transcript deposited in the NCBI database.

**Fig. 4:**
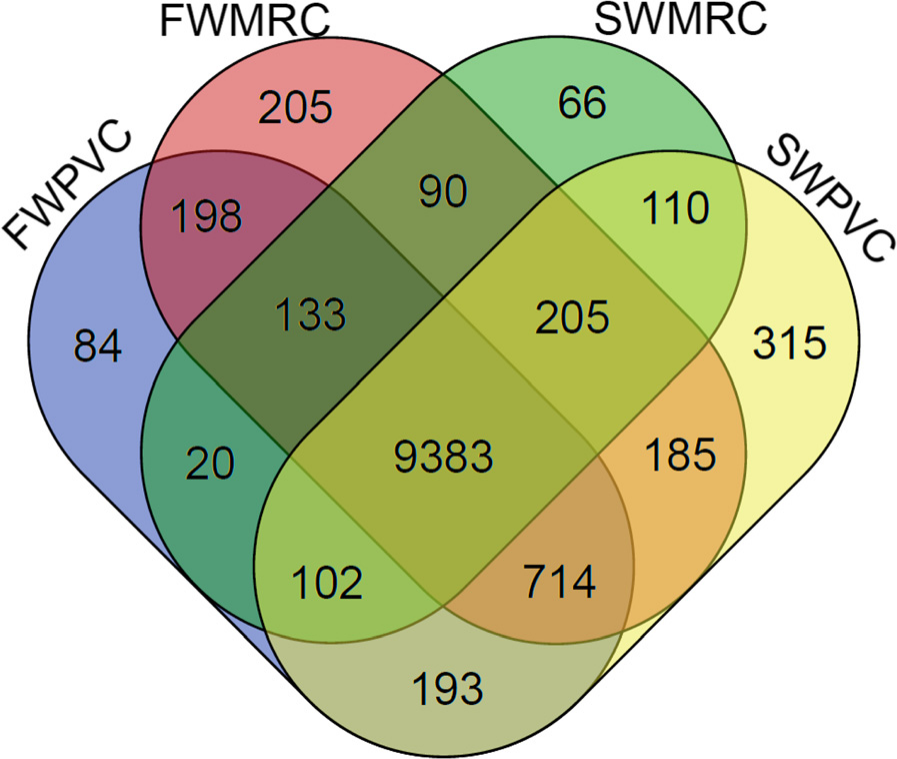
Comparison of annotated genes in FW PVCs, FW MRCs, SW PVCs, and SW MRCs in eel gills. Of the identified genes, 10,392 transcripts were expressed in PVCs, while 9,811 transcripts were expressed in MRCs

**Fig. 5:**
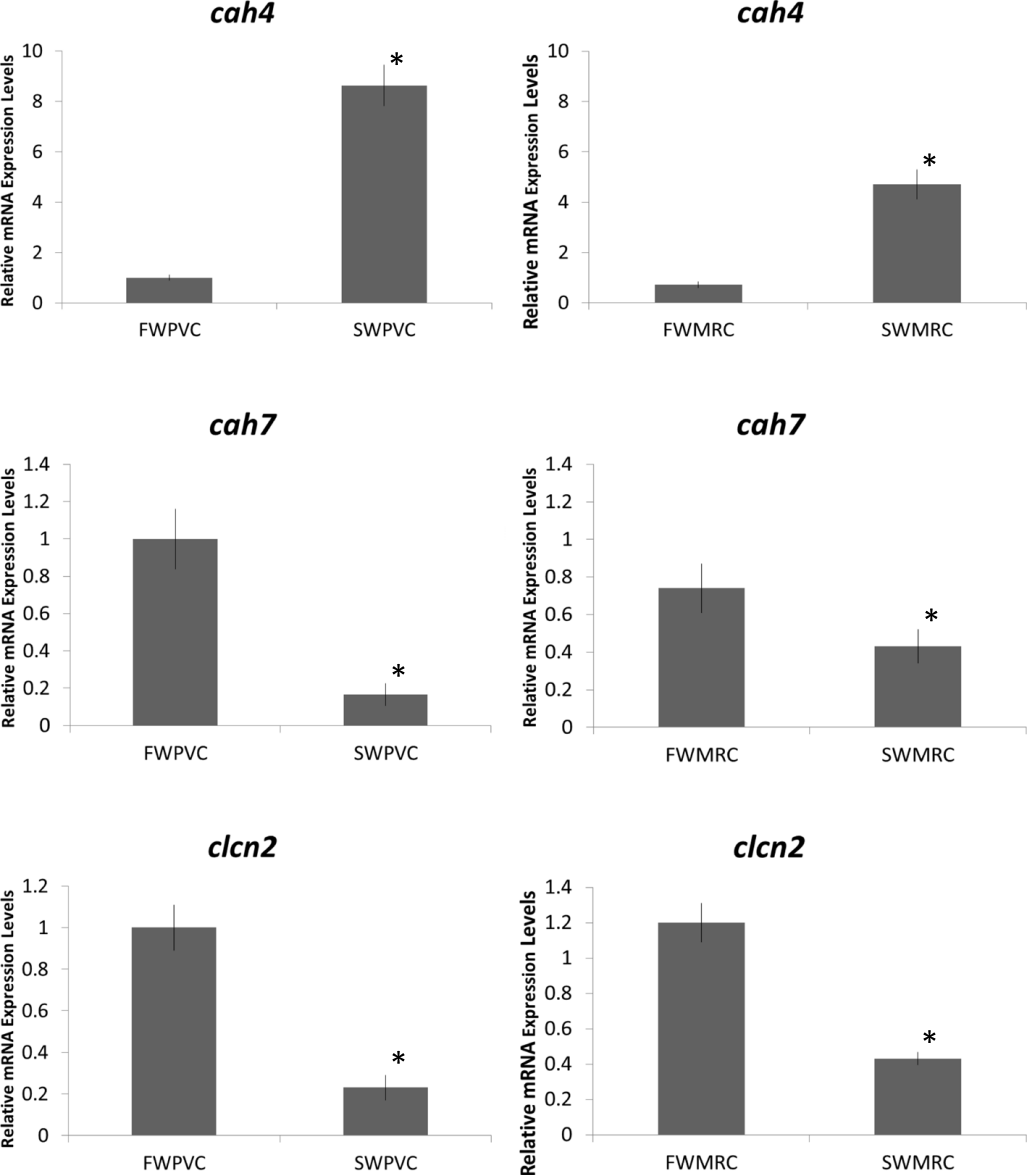
Quantitative real-time PCR of three selected transcripts. Three transcripts were selected. Carbonic anhydrase 4 (*cah4*) was highly expressed in SW gill cells, while carbonic anhydrase 7 (*cah7*) and chloride channel 2 (*clcn2*) were highly expressed in FW gill cells. * indicated *P* < 0.05. All data matched with the RNA-seq result

In Fig. 4, a Venn diagram shows the relations among the four PVC and MRC datasets. In PVCs, there were 10,392 transcripts expressed in both FW and SW conditions. While in MRCs, 9,811 transcripts were commonly expressed in both conditions. Three differential expressed genes, two isoforms of carbonic anhydrase (CA: *cah4* and *cah7*) and one chloride channel (*clcn2*) commonly expressed in both PVCs and MRCs were chosen for validation. The results of the quantitative polymerase chain reaction (PCR) analysis supported the transcriptome data in which FW PVCs and FW MRCs showed greater expression levels of *clcn2* and *cah7,* but lower expression levels of *cah4* compared to the corresponding SW samples (Fig. 5). CLCN2, a chloride channel, belongs to the CLC family of chloride channel/transport proteins. In humans, CLCN2 has been suggested to function in the chloride efflux pathway [26] and its mutation is linked to a central nervous system disorder [27]. While in zebrafish*, clcn2* is suggested to take part in chloride uptake with the apical sodium chloride cotransporter 2b expressing cells, and highly expressed in the MRCs [28, 29]. The greater expression of *clcn2* in FW gill cells might play a role in Cl^−^ transport. The two other validated transcripts, *cah4* and *cah7*, are protein products known to catalyze the inter-conversion of carbon dioxide and water to bicarbonate and protons. CA is recognized to play important roles in gaseous exchanges and the acid–base balance, cooperating with other transporters such as sodium hydrogen exchangers (NHEs) in gills of both FW and SW teleost fish [30–33]. Besides, studies have showed that *clcn2* was highly expressed in MRCs than PVCs in zebrafish [34]. However, no significant difference was found in the eel gill cells, which may due to the species differences. Nevertheless, we have identified the two CA isoforms differentially expressed in FW and SW environments in this study. The importance of the two isoforms in gaseous exchange and acid–base balance or other functions in FW and SW conditions warrants further investigation (Fig. 5).

### Differential expressed genes in PVCs in FW and SW conditions

To analyze changes in molecular responses in individual cell types, the datasets were processed by comparing the transcriptome of each cell type in FW and SW conditions. Bioinformatics analyses using DAVID and IPA were implemented.

**Fig. 6:**
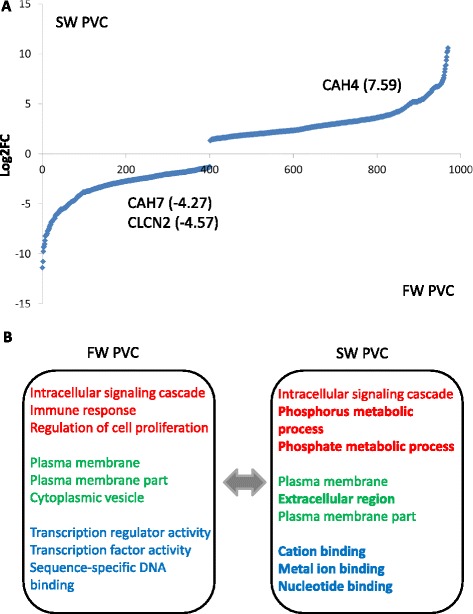
Summary of the DEGs in FW and SW PVCs. **a** 401 transcripts expressed higher in FW and 569 transcripts expressed higher in SW. Selected transcripts for validation are indicated. **b** The top three enriched GO terms in FW PVCs and SW PVCs. BP appears in red; CC appears in green; MF appears in blue. Bolded text indicates the unique GO terms in FW and SW

In comparing the transcriptome data of PVCs in FW and SW conditions, 970 DEGs were found. Among those, 401 transcripts were up-regulated and 569 transcripts were down-regulated in FW PVCs as compared to SW PVCs (Fig. 6a; Additional file 4). The highly expressed transcripts detected in FW PVCs were subjected to gene ontology (GO) analysis. The top three categories in the biological process (BP), including (a) intracellular signaling cascade (21 counts), (b) immune response (20 counts), and (c) regulation of cell proliferation (14 counts), were prioritized. For the cellular component (CC), the top three enriched categories were (a) plasma membrane (55 counts), (b) plasma membrane part (34 counts), and (c) cytoplasmic vesicle (11 counts). For the molecular function (MF), the top three categories were (a) transcription regulator activity (23 counts), (b) transcription factor activity (22 counts), and (c) sequence-specific DNA binding (17 counts) (Fig. 6b; Additional file 5). A GO analysis was also conducted using the transcripts that were highly expressed in SW PVCs as compared to FW PVCs. Noticeably, more GO terms were established in the SW PVCs (290 v. 122 enriched GO terms), suggesting that the additional enriched gene transcripts participated in specific acclimated functions in the SW environment. In the BP, the top three categories were (a) intracellular signaling cascade (27 counts), (b) phosphate metabolic process/phosphorus metabolic process (21 counts), and (c) regulation of cell proliferation/cell adhesion/biological adhesion (19 counts). In the CC, the top three categories were (a) plasma membrane (58 counts), (b) extracellular region (45 counts), and (c) plasma membrane part (29 counts). In the MF, (a) cation binding (65 counts), (b) metal ion binding (64 counts), and (c) nucleotide binding (39 courts) were the top three in the list (Fig. 6b). In comparison with all 412 GO terms between the FW and SW PVCs, there were only 26 common enriched GO terms (marked in yellow in Additional file 5). Interestingly, in the MF, the insulin-like growth factor (IGF) binding activity and growth factor binding activity were enriched in the SW PVCs. The two binding activities are known to be important in SW acclimation [35]. The GO terms cytoskeletal protein binding and calcium ion binding were also enriched in the SW PVCs, suggesting that cytoskeleton reorganization and calcium ion regulation are essential in SW PVCs. Cytoskeletal reorganization is known to be induced in gill cells upon a hyperosmotic challenge while an increase of Ca^2+^ binding may facilitate Ca^2+^ transport and reduce Ca^2+^-induced cytotoxicity in SW environments [18, 36]. Moreover, in the BP, an activation of mitogen-activated protein kinase (MAPK) activity was identified as an enriched GO term in the SW PVCs. This is consistent with our previous studies in indicating the involvement of MAPK cascade in hyperosmotic stress-elicited signaling [37, 38]. Lastly, numerous biosynthetic and metabolic regulatory processes were enriched in SW PVCs (Additional file 5), supporting the previous findings of greater metabolic rates detected in gills of SW fish [39].

### Differential expressed genes in MRCs in FW and SW conditions

**Fig. 7:**
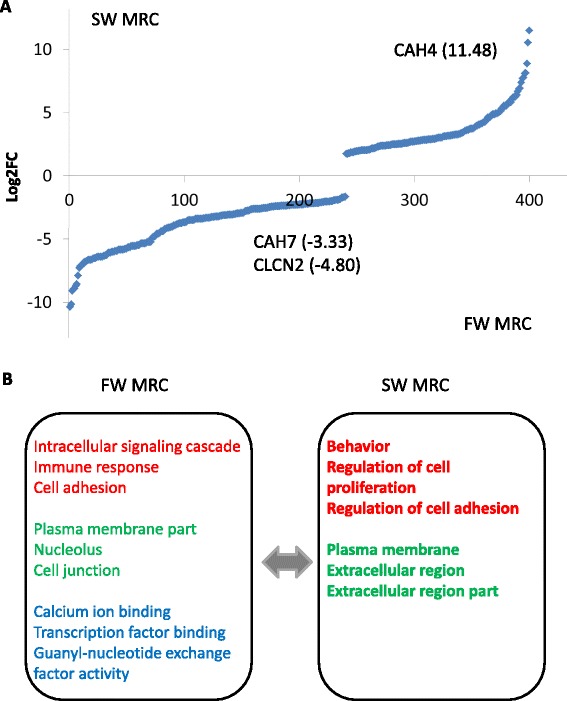
Summary of the DEGs in FW and SW MRCs. **a** 241 transcripts expressed higher in FW and 159 transcripts expressed higher in SW. Selected transcripts for validation are indicated. **b** The top three enriched GO terms in FW MRCs and SW MRCs. BP appears in red; CC appears in green; MF appears in blue. Bolded text indicates the unique GO terms in FW and SW

In the MRCs, 240 transcripts were up-regulated and 160 transcripts were down-regulated in FW conditions as compared to the cells in SW conditions (Fig. 7a; Additional file 6). In the GO analysis, the top three categories under the BP in the FW MRCs were (a) intracellular signaling cascade (15 counts), (b) immune response (11 counts), and (c) cell adhesion/biological adhesion/cell death/death (8 counts). In the CC, the top three categories on the list were (a) plasma membrane part (21 counts), (b) nucleolus (8 counts), and (c) cell junction (7 counts). In the MF, (a) calcium ion binding (9 counts), (b) transcription factor binding (6 counts), and (c) guanyl-nucleotide exchange factor activity/transcription coactivator activity (4 counts) were the three enriched categories (Fig. 7b). While in the SW MRCs, (a) the regulation of cell proliferation/behavior (5 counts) and (b) regulation of cell adhesion/positive regulation of catalytic activity/positive regulation of molecular function (4 counts) were the top enriched categories in BP. (a) The plasma membrane (19 counts), (b) extracellular region (17 counts), and (c) extracellular region part (10 counts) were the highest on the list under the CC. Notably no MF category was identified in the SW MRCs (Fig. 7b). Collectively, the enriched GO terms in FW MRCs were 45, while in SW MRCs were 28. All identified GO terms were unique in the FW and SW conditions (except “behavior” in BP; marked in yellow in Additional file 7).

Collectively for both cell types in FW and SW conditions, PVCs showed more DEGs and had six-fold more GO terms than the MRCs. The data implied that a greater molecular change was required to modify the functional phenotypes of PVCs in waters of different salinities. The result is not surprising as the PVCs cover more than 90 % of the outermost layers of fish gills that are in direct contact with water [14].

### Differential expressed genes in PVCs and MRCs in FW or SW

To provide comprehensive data for readers of this report to identify their own targets, we further compared the PVCs and MRCs in FW or SW conditions. One hundred forty-five DEGs were identified in FW conditions, in which 105 transcripts showed higher expressions in MRCs and 40 transcripts were highly expressed in PVCs. While in SW conditions, there were 1439 DEGs in comparison between PVCs and MRCs. Five hundred forty-six transcripts were highly expressed in SW MRCs, while 893 transcripts were expressed higher in SW PVCs (Additional file 8). Highly expressed SW PVC transcripts involved in tight junction and cytoskeleton regulation were selected and real-time PCR was performed for data validation (Additional file 9). The results suggested that in the SW environment, PVCs actively regulate their intra- and inter-cellular architectures to compensate for the SW environment.

### PVCs and MRCs showed different osmo-responsive signaling pathways against osmotic challenges

**Fig. 8:**
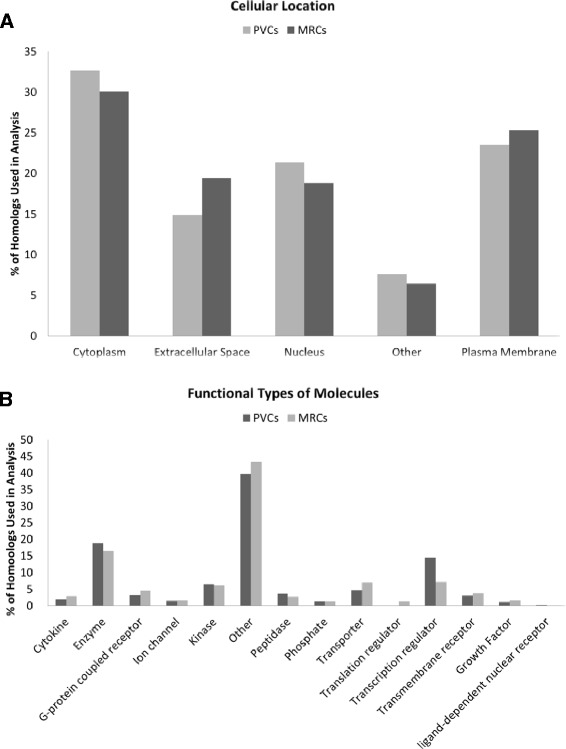
Functional annotation analyses of the eel gill PVCs and MRCs. **a** Distribution (percentage of homologs used in analysis) of the putative human homolog encoded proteins with different sub-cellular locations. **b** Distribution of the putative human homolog encoded proteins according to their functional type

**Fig. 9:**
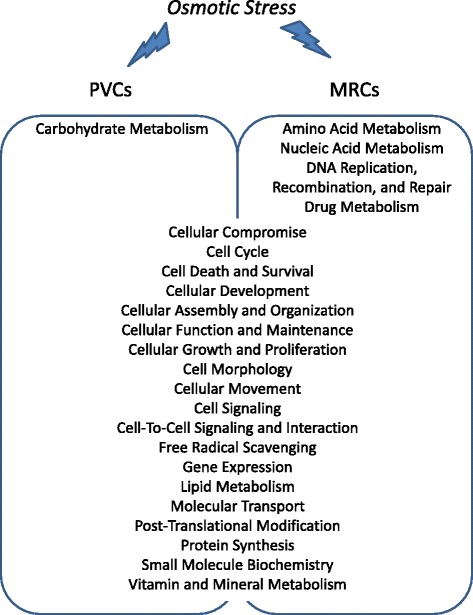
Schematic figure showing the biological functions of the eel gill PVCs and MRCs under osmotic stress. Both PVCs and MRCs achieve similar biological cellular function under osmotic stress

In the latter part of the analysis, IPA software was used to provide an overview of canonical pathways and cellular functions of the DEGs identified in PVCs or MRCs in both FW and SW conditions (using all the DEGs in PVCs or in MRCs). The cellular locations of the DEGs in PVCs and MRCs were defined. Our results showed that 30 % of the DEGs, either identified in PVCs or MRCs, were localized in “cell cytoplasm” (Fig. 8a). Based on the analysis of functional types of molecules, the two major functional categories (excluding the category “other”) observed in both PVCs and MRCs was “enzyme” (18.81 % in PVCs and 16.4 % in MRCs), and “transcription regulator” (14.4 % in PVCs and 7.26 % in MRCs) (Fig. 8b). We further used the DEGs from PVCs and MRCs to perform the enriched biological function analysis. The data clearly demonstrated that the DEGs in both PVCs and MRCs showed similar cellular functions (Fig. 9; Additional file 10). The number of enriched biological functions in PVCs and MRCs were 20 and 23, respectively, in which 19 of them were in common. PVCs and MRCs were found to participate in different metabolic paths. Carbohydrate metabolism was dominant in PVCs while amino acid and nucleic acid metabolism were prevalent in MRCs. More metabolisms were enriched in the MRCs, which might due to the rich mitochondria content in MRCs. In eels, studies on changing metabolism after treatments were performed in the 1970s [40–42]; however, to our knowledge, there are no studies that specifically focus on the metabolism of the two gill cell types. The suggested data here further supports the combined use of advanced metabolomics studies and the measurement of metabolites in gills to understand the underlying metabolism during osmoregulation.

**Fig. 10:**
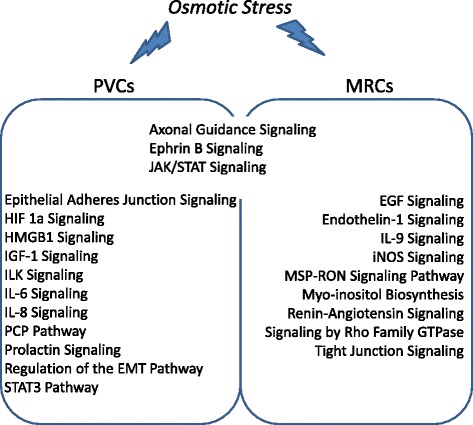
Schematic figure showing the enriched canonical signaling pathways of the eel gill PVCs and MRCs under osmotic stress. PVCs and MRCs enriched different pathways under osmotic stress

In the enriched pathway analysis, some common and cell-type specific signaling pathways were identified to be activated in PVCs and MRCs during osmotic stress (Fig. 10; Additional file 10). The Janus kinase (JAK)/signal transduction and transcription (STAT) factors, axonal guidance, and Ephrin-B were the common signaling pathways identified in both PVCs and MRCs. The JAK/STAT signaling plays an important role in immunity [43], while our previous proteomics analysis in eel gills underlined the expression of different immune-related proteins upon osmotic stress [44, 45]. The enriched pathway supports the notion that the immune system is involved in fish gill osmoregulation. For the axonal guidance signaling, it is mostly associated with cellular growth, proliferation, and development, and is regulated by the Ephrin-B pathway [46, 47]. Gill tissues are known to exhibit substantial alterations in epithelial structures and functions in response to changes in water salinity and hardness [35]. This axonal guidance/Ephrin-B pathway may contribute to the proliferation and anatomical localization of PVCs and MRCs to induce functional plasticity of gill tissues to withstand osmotic challenges. In this regard, our finding supports the hypothesis on the “short-term transformation and long-term replacement” in describing gill tissue remodeling processes during early and later phases of acclimation at different water salinities [48].

In addition to the three common signaling pathways, other cell-type specific pathways were identified. PVCs were found to be regulated by two important hormonal pathways, the prolactin signaling and IGF-1 signaling. Prolactin was reported to reduce ion leakage in gills and to play an important role in FW acclimation [35]. It has recently been suggested that prolactin could regulate the expression of the sodium chloride cotransporter via prolactin receptor in zebrafish gills [49, 50]. The IGF-1 pathway was shown to be involved in SW acclimation [51, 52]. Moreover, our previous study showed that PVCs were responsive to prolactin or IGF-1 treatment to increase the expression levels of the Na^+^/K^+^/Cl^−^ co-transporter (NKCC) [53]. In addition to hormonal signaling, the integrin-linked kinase (ILK)-Akt (protein kinase B), cytokine-related pathways such as IL6, and IL8 signaling pathways and hypoxia-inducible factor-1α (HIF-1α) pathways were identified in PVCs. These pathways have been well studied in human carcinogenesis [54–56], but their roles in osmoregulation remain unclear. In addition, the IPA identified the epithelial adherens junction signaling and epithelial mesenchymal transition (EMT) regulatory pathway in PVCs. These identified processes are highly related to cell proliferation/migration, cell-cell interaction, and functional transformation. Cell migration was reported during gill remodeling in fish acclimated in waters of different salinities and hardness [21, 57–59]. In fact, an activation of the Akt pathway was shown to induce the expression of osmotic stress transcription factor 1 (Ostf1) during hyperosmotic stress in a PVC culture [44]. The osmoregulatory cytokines, such as tumor necrosis factor α (TNF-α) and interleukins (ILs), were reported to play roles in the osmotic stress signaling network in fish to regulate epithelial responses to salinity changes [58, 60–62].

For MRCs, two other cytokines-related pathways, IL9 and endothelin-1 (EDN1), were recognized. MRCs are well-known for their role in chloride transport [4]. Exogenous EDN1 was reported to regulate chloride transport in the opercular epithelium of killifish; its mRNA expression levels were demonstrated to be increased in gills of killifish during SW acclimation [63]. Moreover EDN1 was recently suggested to inhibit the type A natriuretic peptide receptor, to reduce the expression of nitric oxide synthase (NOS), and to regulate the urinary sodium concentration in rat inner-medullary collecting duct cells [64, 65]. In this regard, NOS was shown to inhibit the expression of sodium pump (Na^+^/K^+^-ATPase) in the gills of salmon and trout [66, 67]. Since the MRCs are featured with a high abundance of Na^+^/K^+^-ATPase, an understanding of the functional roles of EDN1 and NOS in the regulation of Na^+^-transport in MRCs for osmoregulation is important. In addition, some well-known osmoregulatory pathways like epidermal growth factor (EGF) signaling and renin-angiotensin (RA) signaling were also identified. The EGF signaling pathway is involved in volume and ion regulation [68–70], while the RA signaling is important for epithelial Na^+^ transport and pressor functions [71, 72]. Furthermore, the myo-inositol biosynthesis pathway for the synthesis of a universally compatible osmolyte, myo-inositol [73–77], was found to be enriched. Lastly, the analysis identified the tight junction signaling and Rho GTPase signaling, and the osmotic stress induced Rho pathway has been related to the tight junction rearrangement [78]. It should be noted that the enrichment of a particular pathway is based on the bioinformatics analysis. The DEGs during osmotic stress do not cover all the transcripts that are involved in the pathway, and they can be up- or down- regulated. To reach a solid conclusion, researchers must perform further experiments on specific targets.

#### Conclusion

Physiological and molecular functions of gill cells have been studied for more than 60 years; however, the molecular responses in the gill PVCs and MRCs in relation to their functional phenotypes remain unclear. Thus, we applied transcriptomic analysis to identify differential expressed transcripts in the two major gill cell types (PVCs and MRCs) in FW- and SW- acclimated eels. The study identifies newly annotated transcripts to enrich the eel gill database, and, for the first time, shows specific osmoregulatory adaptive responses in the PVCs and MRCs.

We understand that there may be a mis-annotation of eel sequences if using the other available model species databases. Although there are published draft eel genome databases available, such as EeelBase and ZF-Genomics, the annotated sequences and confidence reads are still relatively small compared to other model organisms, which have limited the use of these databases for BLAST sequencing. There is a lack of integrated information for accurate bioinformatics prediction. Efforts must be made to facilitate data exchange and communication between different groups after release of a dataset. The presence of highly similar isoforms, or unique eel transcripts could hamper the recent transcriptomic analysis studies; we are looking forward to the completion of the eel genome, which provides the real genome reference of this economically valuable and endangered species.

## Method

### Animals and isolation of gill cells

Japanese eels (*Anguilla japonica*) weighing 500–600 g were purchased and kept in two 40 L glass tanks supplied with charcoal-filtered aerated FW or SW (34 ppt) at 18–20 °C under a 12 L:12D photoperiod. Ten liters of water was changed every 3 days. The fish (*n* = 4) were sampled after 3 weeks. The fish were anesthetized by 0.1 % MS222 in a plastic container and the gills were perfused with buffered saline (130 mM NaCl, 2.5 mM KCl, 5 mM NaHCO_3_, 2.5 mM glucose, 2 mM EDTA, and 10 mM Hepes, pH 7.7) to remove blood cells from gills. Gill arches were excised and washed. The gill arches were cut into small fragments and subjected to two cycles of trypsin digestion (0.5 % trypsin + 5.3 mM EDTA), each for 20 min at room temperature in a rotator (300 rpm). The cell suspension was then filtered, washed, and underwent a three-step Percoll gradient of 1.09, 1.06, and 1.03 g/ml in PBS and centrifuged at 2,000 g at 15 °C for 20 min. Cells at the interface of 1.03 and 1.06 g/ml Percoll solution were regarded as PVCs, while at the interface of 1.06 and 1.09 g/ml were MRCs. The identity of MRCs was confirmed by mitochondria staining (Mitotracker, CMTMRos- H2, Molecular Probes) and Na^+^/K^+^-ATPase staining (mouse anti-Na^+^/K^+^-ATPase á-subunits antiserum) (1:10, Developmental Studies Hybridoma Bank, the University of Iowa) [21, 38, 79]. The methods were carried out in Hong Kong Baptist University in accordance with the approved guidelines. All experimental procedures were approved by the Hong Kong Baptist University, Hong Kong.

### Library construction and Illumina RNA-seq

The overall workflow of the report is shown in Fig. 1. Briefly, total RNA from FW PVCs, FW MRCs, SW PVCs and SW MRCs (each *n* = 2) was extracted using the mirVanaTM miRNA isolation kit (Applied Biosystems). RNA quality was assessed using the Agilent 2100 Bioanalyzer system and samples with a RNA Integrity Number (RIN) greater than 8 were used for RNA library construction. The cDNA libraries were constructed according to manufacturer’s instruction and index codes were ligated as identification to individual samples as previous described. Briefly, 8 cDNA libraries were constructed (2 X 4 groups), each prepared from 300 ng of total RNA. mRNA was purified from the total RNA using poly-T oligo-attached magnetic beads (Illumina, San Diego, USA) to remove the ribosomal RNA. Then the mRNA was fragmented by using divalent cations in Illumina proprietary fragmentation buffer at 94 °C for 1 min. First strand cDNAs were synthesized using random oligonucleotides and SuperScript II, then second cDNAs were synthesized using DNA polymerase I and RNase H. Overhangs were blunted by using exonuclease/polymerase, followed by 3’ end adenylation. After adenylation of 3’ ends of DNA fragments, Illumina PE adapter oligonucleotides were ligated to DNA fragments. DNA fragments that ligated with adaptor molecules on both ends were selectively enriched using Illumina PCR Primer Cocktail in a 15 cycles PCR reaction. Libraries were purified using AMPure XP system and quantified using the KAPA Library Quantification Kits. Before submitted to sequencing, the libraries were normalized and pooled together in a two single lane on an Illumina MiSeq platform and 150 bp paired-end reads were generated. Adapters and reads containing poly-N were first trimmed and the sequence-reads were dynamically trimmed according to BWA’s - q algorithm [80]. Briefly, a running sum algorithm was executed in which a cumulative area-plot is plotted from 3’-end to the 5’-end of the sequence reads and where positions with a base-calling Phred quality lower than 30 cause an increase of the area and vice versa. Such plot was built for each read individually and each read was trimmed from the 3’-end to the position where the area was greatest. Read-pairs were then synchronized such that all read-pairs with sequence on both sides longer than 35 bp after quality trimming were retained. Any singleton read resulting from read trimming was removed [80]. All the downstream analyses were based on quality trimmed reads.

### De novo Transcriptome assembly

Forward and reverse reads from all the libraries/samples were pooled and subjected to transcriptome de novo assembly using Trinity (version r20140413p1) with “min_kmer_cov” set to 2; “SS_lib_type” set to RF, and all other parameters set to default [81]. Trinity uses fixed k-mer to generate an assembly and it is efficient in recovering full-length transcripts as well as spliced isoforms.

### Annotation of assembled transcripts

Coding sequences (open reading frames, ORF) were identified by Transdecoder [82] using the following criteria: (1) the longest ORF was identified within each transcript; (2) from the longest ORFs extracted, a subset of the longest ones was identified and randomized to provide a sequence composition corresponding to non-coding sequences before being used to parameterize a Markov model based on hexamers; and (3) all the longest ORFS were scored according to the Markov Model to identify the highest scoring reading-frame out of the six possible reading-frames. These ORF were then translated to protein sequences and subjected to (1) BLASTp search against UniProtKB/Swiss-Prot with a cut-off e-value [83, 84] of 1.0 × 10^−6^, (2) protein domain search via HMMScan, (3) transmembrane helicase prediction by TMHMM, and (4) signal peptide prediction by SignalP.

### Comparative analysis of 454 and MiSeq assembled eel gill transcriptome

The eel gill transcriptome of whole gill samples generated by 454 platform was obtained [17]. The transcripts generated by MiSeq and 454 were considered to be the same transcript if they were the symmetrical best hits in each reciprocal all-against-all BLASTn search (i.e. Reciprocal Best Hit [85]). Briefly, putative match to the 454 sequences were identified first by comparing the MiSeq assembled transcript to the 454 database using BLASTn search. The highest-scoring hit was obtained and, then, a BLASTn search was run against the database of the assembled transcripts. The hit in 454 sequences was considered an identical transcript of the assembled transcript if and only if the second BLAST search returned the assembled transcript that was the highest scorer in the first BLAST search. mRNA transcripts with complete coding sequence of Anguilla species were retrieved from NCBI using search term (Anguilla japonica[porgn] AND (complete[All Fields] AND cds[All Fields])).

### Differential expression, GO and pathway enrichment analysis

Differential gene expression and TMM-normalized FPKM gene expression were calculated by RSEM pipeline using edgeR package [86]. Samples with identical treatments were considered to be biological replicates. Genes with B&H corrected *p*-value <0.05 and log2 (fold change) >1 were considered be statistically significant differentially expressed. The Database for Annotation, Visualization and Integrated Discovery (DAVID) was used for functional annotation clustering analysis on the all samples comparison (FW PVCs Vs SW PVCs/FW MRCs Vs SW MRCs) with the classification stringency as Benjamini-Hochberg corrected *P*-value (*P* < 0.05) [87]. The dysregulated transcripts (FW PVCs Vs SW PVCs and FW MRCs Vs SW MRCs) with human homologs of the assembled contigs were then underwent the IPA software to identify functional canonical pathways and functions (www.qiagen.com/ingenuity) with the significance level set at Benjamini-Hochberg corrected *P*-value (*P* < 0.05).

### Real-time PCR analysis

The isolated enriched PVCs or MRCs were dissolved in Tri-Reagent (Gibco-BRL) for total RNA extraction. Total RNA with a ratio of 1.8–2.0 at A260/A280 was used for cDNA synthesis. Briefly, 0.5 μg total cellular RNA was reverse transcribed by VILO (Invitrogen). PCR was conducted using the Applied Biosystems 7500 real-time PCR detection system using KAPA SYBR® Green Supermix (KAPA). Primers used in the real-time PCR assay were designed on the basis of transcriptome sequence. Sequences of the real-time PCR primers were tabulated in Additional file 11. The data were then normalized using the expression levels of *gapdh* mRNA [88]. The existence of primer–dimers and secondary products was checked using melting curve analysis. Our data indicated that the amplification was specific. Only one PCR product was amplified for each individual primer set. The relative expression ratio was calculated according to the method described by Pfaffl [89].

### Availability of supporting data

The sequencing data from this study have been submitted to the NCBI Sequence Read Archive (SRA) (http://www.ncbi.nlm.nih.gov/ sra) under the accession number SRP049703. Other data supporting the result of this article are included within the article and the Additional files 1, 2, 3, 4, 5, 6, 7, 8, 9, 10, 11.

## Additional files

Additional file 1:
**List of 12,003 identified transcripts in eel gill cells.** (XLSX 28990 kb) Additional file 2:
**Taxonomic distribution of the 12,003 identified eel gill transcripts.** (XLSX 9 kb) Additional file 3:
**Comparison of the present cell-type specific transcriptome data to our previous whole-gill tissue transcriptome.** (XLSX 1505 kb) Additional file 4:
**List of the 970 DEGs identified from FW PVCs and SW PVCs.** (XLSX 117 kb) Additional file 5:
**Gene ontology of DEGs identified from FW PVCs and SW PVCs.** (XLSX 29 kb) Additional file 6:
**List of the 400 DEGs identified from FW PVCs and SW PVCs.** (XLSX 51 kb) Additional file 7:
**Gene ontology of DEGs identified from FW MRCs and SW MRCs.** (XLSX 13 kb) Additional file 8:
**List of DEGs identified from FW MRCs and FW PVCs, and SW MRCs and SW PVCs.** (XLSX 173 kb) Additional file 9:
**Real-time PCR of selected SW PVC highly expressed transcripts that contributed to the tight junction and cytoskeleton reorganization.** (PDF 279 kb) Additional file 10:
**Ingenuity pathway analysis of the DEGs identified from PVCs or MRCs.** (PDF 253 kb) Additional file 11:
**Primers for real-time PCR of selected genes.** (XLSX 11 kb)
