# Supplementary material for: Transcriptomic analysis reveals specific osmoregulatory adaptive responses in gill mitochondria-rich cells and pavement cells of the Japanese eel
Source: BMC Genomics. 2015 Dec 18;16:1072. doi: 10.1186/s12864-015-2271-0 (PMC4683740; doi:10.1186/s12864-015-2271-0)
Supplement: Additional file 9: — Real-time PCR of selected SW PVC highly expressed transcripts that contributed to the tight junction and cytoskeleton reorganization. (PDF 279 kb) [file 12864_2015_2271_MOESM9_ESM.pdf]

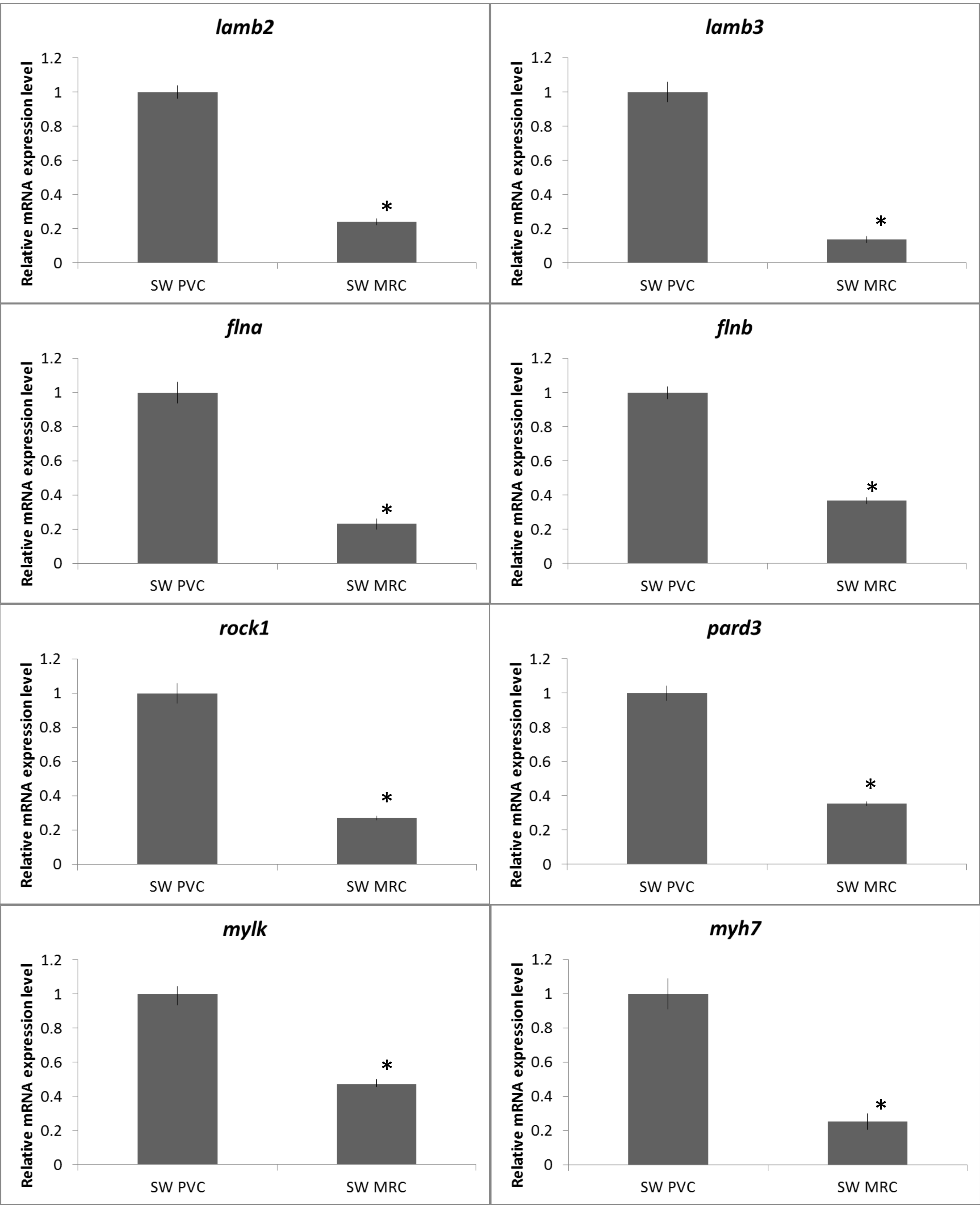

lamb2
lamb3
flna
flnb
rock1
pard3
mylk
myh7
Relative mRNA expression level
0
0.2
0.4
0.6
0.8
1
1.2
SW PVC
SW MRC
*

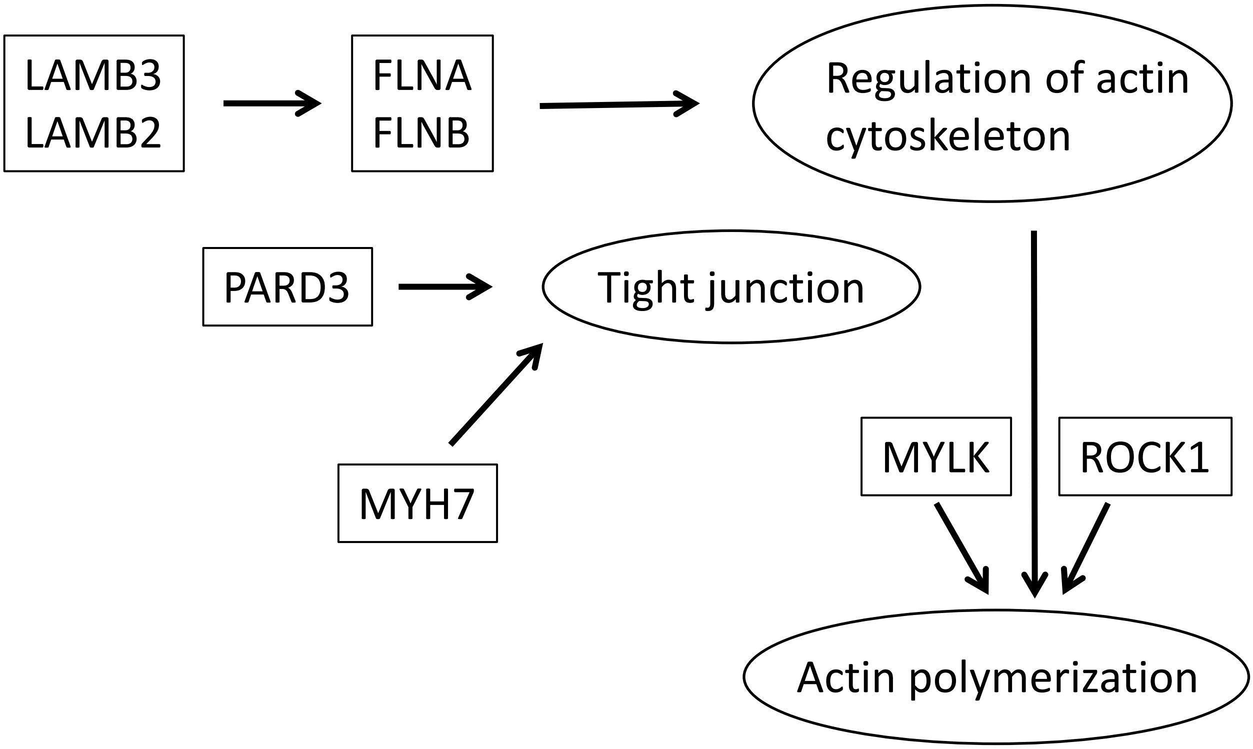

Real-time PCR of selected SW PVC highly expressed transcripts that contributed in tight junction and cytoskeleton regulation. Focal adhesion transcripts such as laminin beta 2 (*lamb2*),laminin beta 3 (*lamb3*), filamin A (*flna*), filamin B (*flnb*), rho-associated protein kinase 1 (*rock1*) and myosin light chain kinase (*mylk*), could stimulate the reorganization of actin cytoskeleton and regulate actin polymerization. Adherens junction transcripts such as par-3 family cell polarity regulator (*pard3*) and myosin 7 (*myh7*) will regulate the tight junction.
