# Supplementary material for: Transcriptomic analysis reveals specific osmoregulatory adaptive responses in gill mitochondria-rich cells and pavement cells of the Japanese eel
Source: BMC Genomics. 2015 Dec 18;16:1072. doi: 10.1186/s12864-015-2271-0 (PMC4683740; doi:10.1186/s12864-015-2271-0)

PVC biological functions

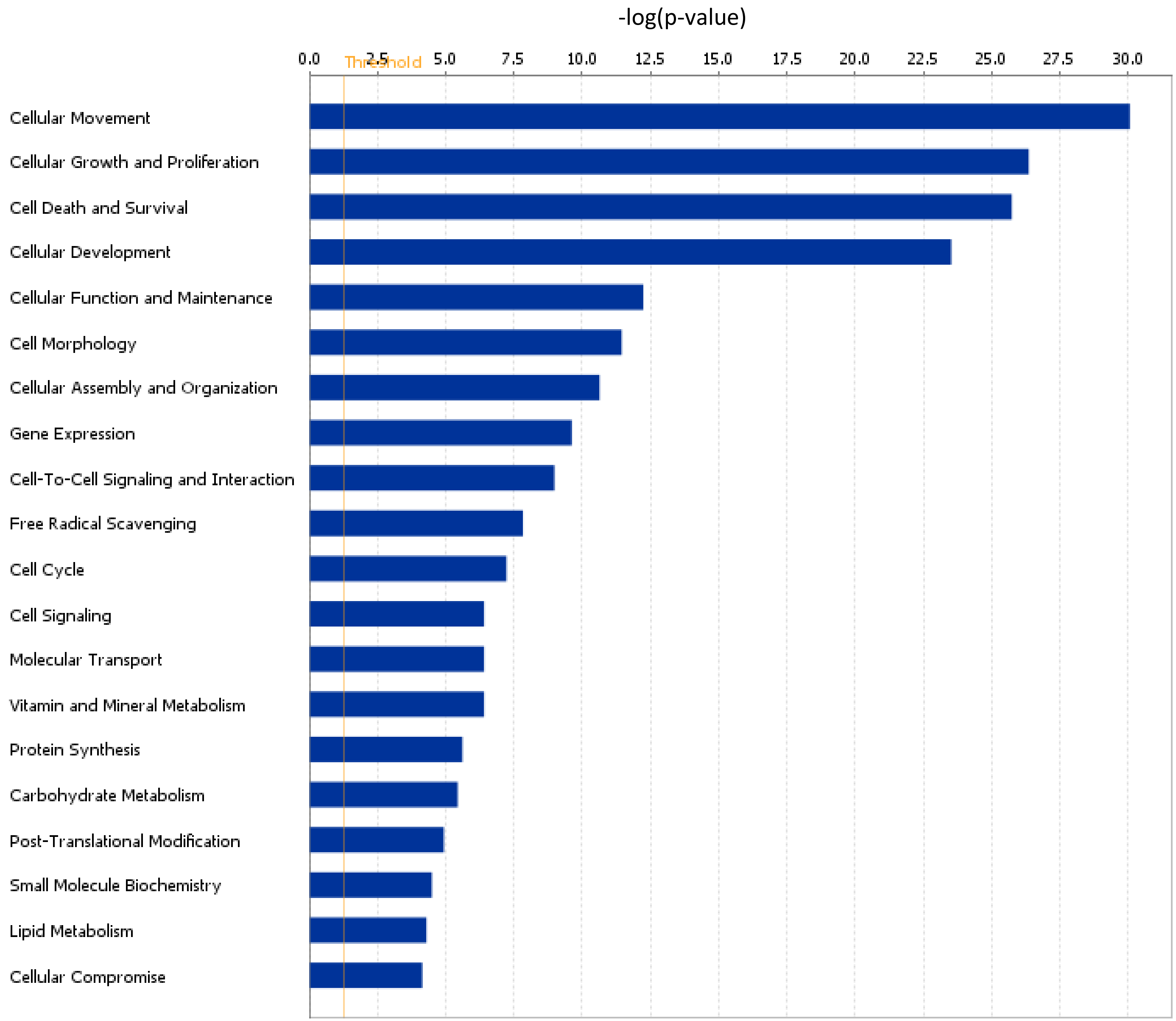

PVC signaling pathways

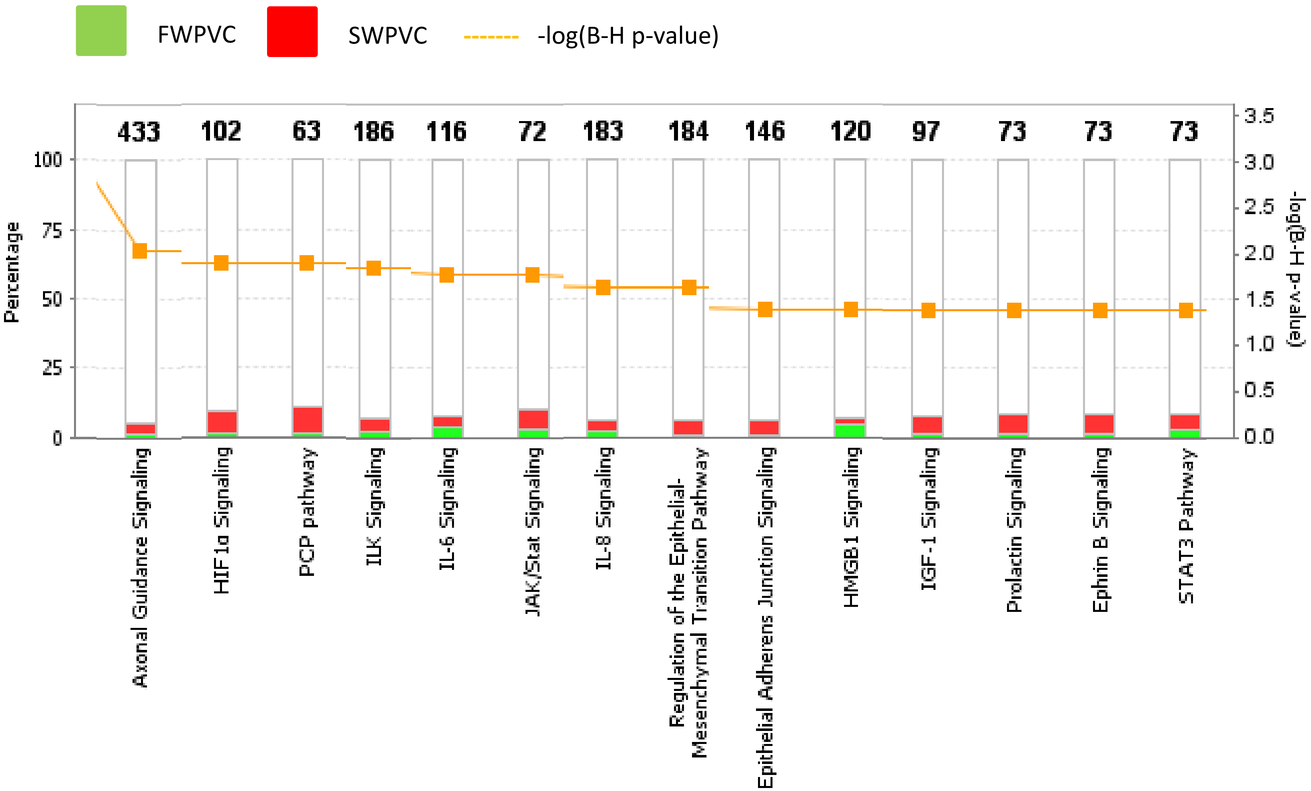

MRC biological functions

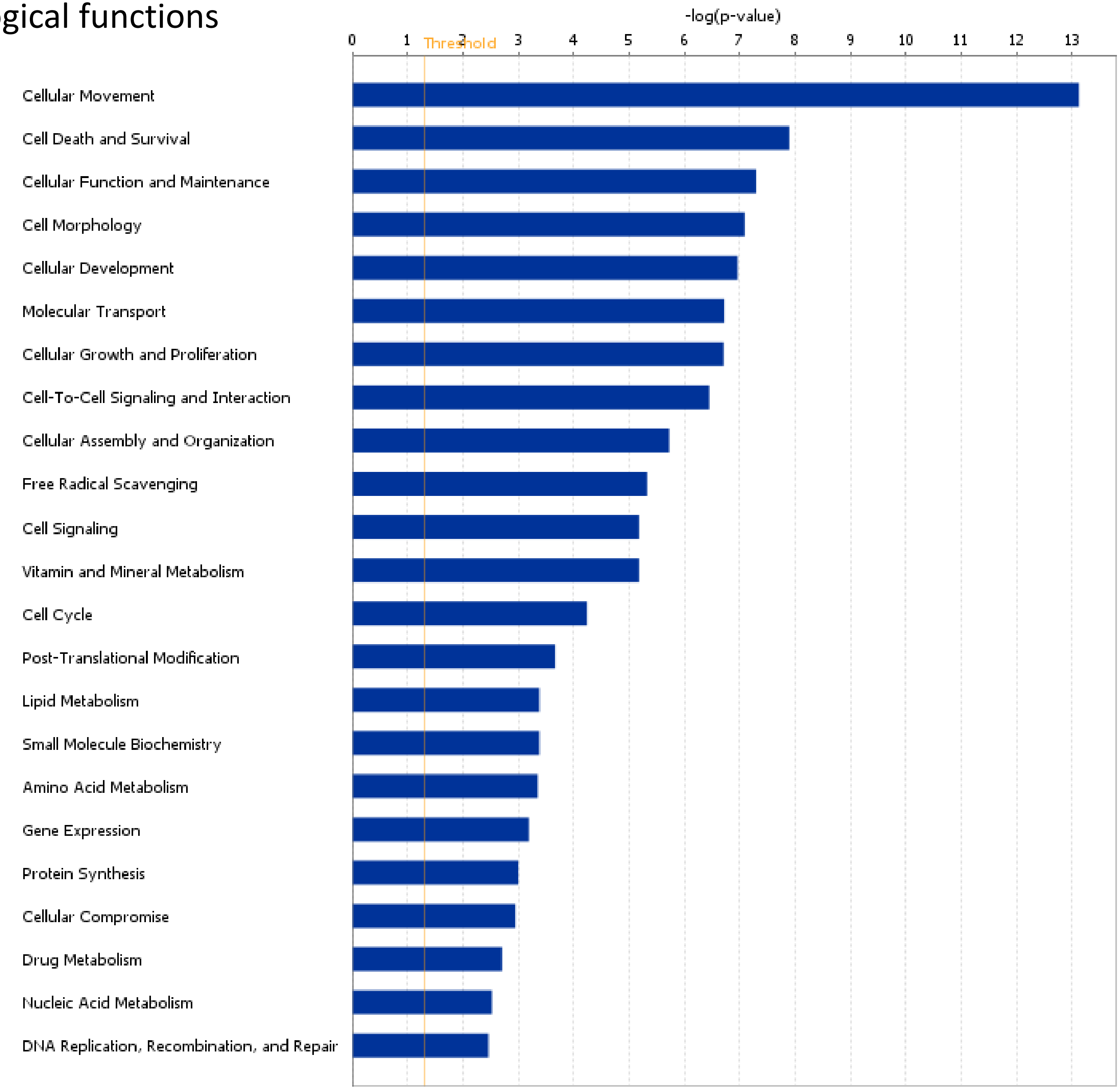

MRC signaling pathways

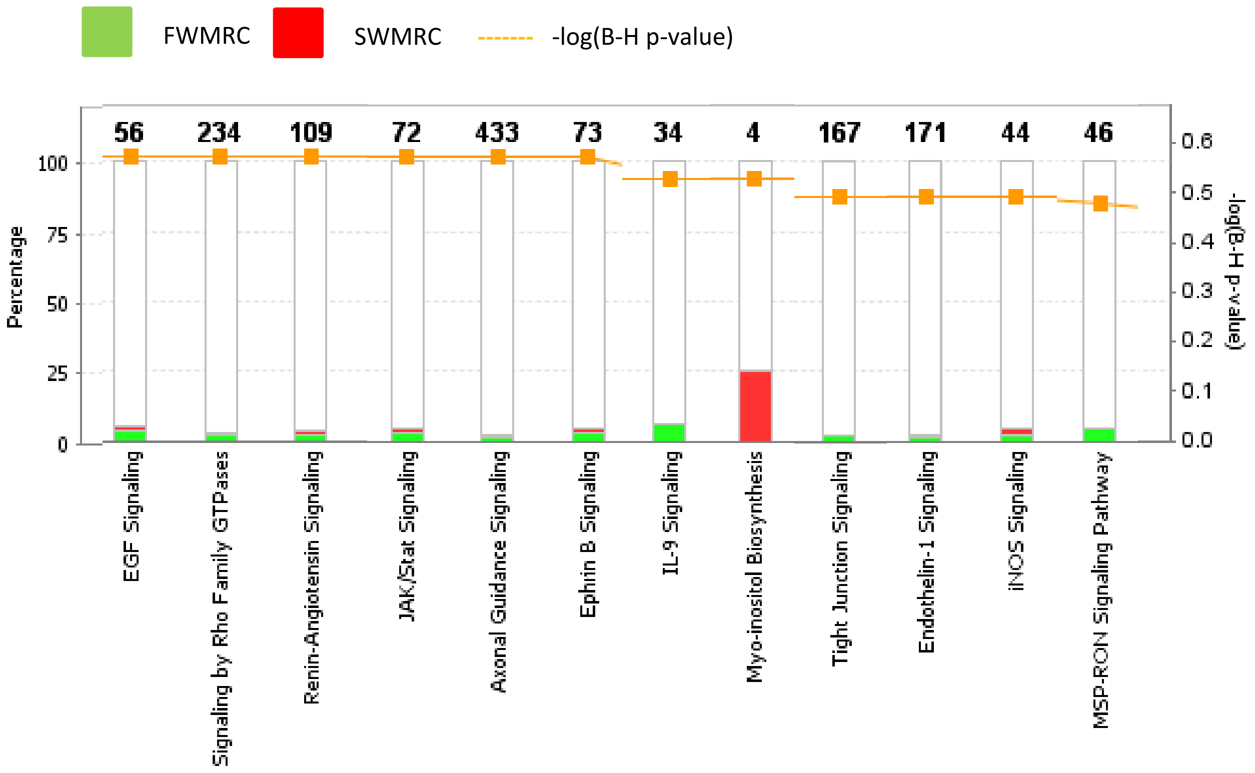

Supplement: Additional file 10: — Ingenuity pathway analysis of the DEGs identified from PVCs or MRCs. (PDF 253 kb) [file 12864_2015_2271_MOESM10_ESM.pdf]
